# Comparing Medical and Surgical Management of Chronic Rhinosinusitis: A Systematic Review of Dupilumab and Endoscopic Sinus Surgery

**DOI:** 10.7759/cureus.79742

**Published:** 2025-02-27

**Authors:** Brandon Weissman, Kevin Shen, Octavia L Flanagan, Shafayath Chowdhury, John Sawires

**Affiliations:** 1 Otolaryngology, Lake Erie College of Osteopathic Medicine, Buffalo, USA; 2 Pharmacology, Lake Erie College of Osteopathic Medicine, Pittsburgh, USA; 3 College of Osteopathic Medicine, Lake Erie College of Osteopathic Medicine, Elmira, USA; 4 Anesthesiology and Critical Care, Lake Erie College of Osteopathic Medicine, Elmira, USA

**Keywords:** chronic rhino-sinusitis, dupilumab, dupixent, endoscopic sinus surgery (ess), functional endoscopic sinus surgery (fees)

## Abstract

Chronic rhinosinusitis (CRS) is a multifactorial condition that significantly impacts patients’ quality of life through persistent nasal obstruction, loss of smell, and facial pain. Endoscopic sinus surgery (ESS) has historically been the definitive option for patients who fail first-line therapies, yet emerging evidence suggests that Dupixent (dupilumab), an IL-4/IL-13 inhibitor, may provide robust medical management by directly targeting the underlying type 2 inflammatory pathways. This systematic review adhered to the Preferred Reporting Items for Systematic Reviews and Meta-Analyses (PRISMA) guidelines, examining randomized clinical trials and prospective intervention studies identified through multiple databases.

Findings indicate that both ESS and Dupixent can substantially improve symptoms and overall quality of life in patients with CRS. Surgical intervention offers rapid anatomical relief of nasal obstruction and may reduce bacterial biofilm prevalence, but improvements in olfactory outcomes are often limited, especially in non-polyp patients. Meanwhile, Dupixent consistently demonstrated significant reductions in nasal polyp burden, SNOT-22 scores, and enhanced olfactory function, particularly in those with pronounced type 2 inflammation. However, questions remain regarding its long-term safety, tolerability, and financial feasibility, given its ongoing nature and high cost.

This review underscores the complementary roles of medical and surgical therapies: Dupixent addresses the inflammatory aspect of CRS, while ESS corrects anatomical blockages and improves local medication delivery. Ultimately, patient-specific factors-including comorbid conditions, severity of polyposis, response to first-line therapies, and cost considerations-should guide treatment selection. Ongoing large-scale trials and head-to-head comparisons will be pivotal in deciding optimal management strategies for chronic rhinosinusitis with nasal polyps (CRSwNP) and refining personalized treatment approaches.

## Introduction and background

Chronic rhinosinusitis (CRS) assessment and management

Chronic rhinosinusitis (CRS) is a condition that can affect many aspects of a patient's life and well-being. Within the United States, it is reported that there are approximately 1,048 cases of CRS per 100,000 people [1]. CRS is characterized by a constellation of patient-reported symptoms, including nasal blockage/obstruction/congestion, anterior and posterior nasal drainage, facial pain/pressure, and a decreased sense of smell. Patients who report two or more of these symptoms for at least three consecutive months are at high risk for having CRS [1]. Patients may experience a wide range of these symptoms or have none and still be diagnosed with CRS. During an endoscopic and physical examination of the patient, objective findings may include nasal polyps, mucopurulent drainage from the middle meatus, edema or mucosal obstruction in the middle meatus, or other structures with sinus outflow. Radiologic examination often reveals mucosal thickening or opacification in the ostiomeatal complex or paranasal sinuses.

SNOT-22 scoring can be used to assess the severity of CRS symptoms. This questionnaire is based on patient-reported symptoms, including severity and quality of life (QoL). The survey uses a Likert scale, which scores the responses from 0 to 5, with 0 indicating no symptoms and 5 being the most severe symptoms [2]. The SNOT-22 can also be used to evaluate the effectiveness of treatment for CRS. This scoring system uses entirely patient-reported side effects, which is subjective in nature.

Another assessment tool for CRS is the Lund-Mackay staging system. This scoring system has been adopted for radiologic evidence and staging of CRS. The system uses CT images of the sinuses to determine whether they are completely clear, partially opaque, or completely opaque [3]. From this, a score is determined. Some surgeons use this score to determine eligibility for surgery, with a score of 4 or above; however, there is no universal cutoff, as there are no guidelines based on this score [3].

Diagnosis of chronic sinusitis includes diagnostic imaging and SNOT-22 scoring. The gold standard is a non-contrast CT scan of the sinuses. CT scans are more sensitive than nasal endoscopy and can provide an anatomical map for surgery [1]. Once diagnosed, CRS is divided into two variants: with nasal polyps or without nasal polyps. It is estimated that one-third of CRS patients have nasal polyps [1]. Pathogenesis is complex and involves a dynamic interplay between host factors (immune response, genetic predisposition, anatomic variations), microbial factors (bacteria, fungi, viruses), and environmental influences (allergens, pollutants) [1]. However, it is determined that certain subtypes of CRS can be divided into type 2 and non-type 2 inflammation. Studies show that the prevalence of these subtypes can vary greatly by geography and clinical context [1]. Type 2 inflammation classically will promote an eosinophil and immunoglobulin E (IgE) response.

Current treatment modalities for chronic sinusitis

The initial guidelines for treating CRS with and without nasal polyps are clear. These guidelines, developed by the American Academy of Otolaryngology-Head and Neck Surgery, suggest that the first step in treating CRS is recommending that the patient begin saline nasal irrigation and/or topical intranasal corticosteroids [4]. If the patient is unsuccessful in relieving symptoms after two weeks of this treatment, the guidelines suggest that the physician counsel the patient regarding medical or surgical management [4].

Medical Management of CRS

Dupixent (Dupilumab) is a fully human-derived monoclonal antibody that blocks the shared receptor component for interleukin-4 (IL) and IL-13, inhibiting the signaling for IL-4 and IL-13 [5]. Both IL-4 and IL-13 are cytokines that play an essential role in immune function. IL-4 helps differentiate naive helper T cells into T helper type 2 (Th2) cells and promotes class switching to IgE in B cells. IL-13 also plays a role in tissue remodeling and helps regulate inflammation. When the body's immune system shifts toward a Th2 response, it becomes active in dealing with allergens or harmless substances, which may lead to an overcompensation that creates severe allergic symptoms such as a runny nose, coughing, sneezing, and other associated symptoms. Dupilumab is indicated for the maintenance treatment of chronic rhinosinusitis with nasal polyps (CRSwNP) in patients 12 years of age and older who fail to respond well to steroid/irrigation treatment.

Surgical Management of CRS

Functional endoscopic sinus surgery (FESS), encompassed within endoscopic sinus surgery (ESS) for the purpose of this review these terms will be used interchangeably, is the most commonly performed surgical approach for treating CRS with and without nasal polyps [6]. The minimally invasive procedure enlarges the sinus ostia, which improves the aeration of the sinuses and enhances mucociliary transport [6]. The objectives of the procedure are to restore ventilation or drainage pathways to enhance the delivery of medications/rinses, remove diseased tissue such as polyps, osteitic bone, and accumulations of mucus or fungal debris, preserve sinus and nasal mucosa to aid in mucociliary clearance and preserve anatomic landmarks to reduce the risk of injury [7]. Recent research on IL-13 levels post-endoscopic surgery for CRS has shown a significant reduction in IL-13 following the procedure, which may help explain the therapeutic effect of surgery on CRS symptoms [8].

Research study aims and questions

This systematic literature review explores two treatment options for managing CRS with and without nasal polyps: medical management with Dupixent and functional ESS. Before Dupixent’s FDA approval in 2019, functional ESS was a common treatment modality for patients with CRS who failed first-line therapy. We aim to answer the question: Is medical management with Dupixent as effective as sinus surgery in managing CRS symptoms and improving QoL? Longitudinal data on health outcomes from the use of Dupixent are currently being gathered through a National Institutes of Health (NIH) grant contract sponsored by Regeneron Pharmaceuticals from June 2021 to July 2027 under the study name study Assessing Long-term Outcomes of dupilumab (DUPIXENT®) Treatment in Adult Patients With Chronic Rhinosinusitis With Nasal Polyposis (CRSwNP) (AROMA); therefore, the literature yields sparse long-term health outcomes following its use. This review is aimed at helping clinicians make decisions on the management of CRSwNP after first-line therapy fails.

## Review

Methodology

Design

This systematic review follows the Preferred Reporting Items for Systematic Reviews and Meta-Analyses (PRISMA) 2020 guidelines and did not require review or approval by the ethics committee.

Search Strategy

A literature search was conducted on PubMed, MEDLINE via Ovid, Embase via Ovid, the World Health Organization-International Clinical Trials Registry Platform, and Clinicaltrials.gov from September 2009 until September 2024 to find articles that may be eligible. A search strategy was employed using specific key search terms, which included "Dupixent" OR "Functional endoscopic sinus surgery" AND "Chronic Rhinosinusitis."

Selection Criteria

**Figure 1:**
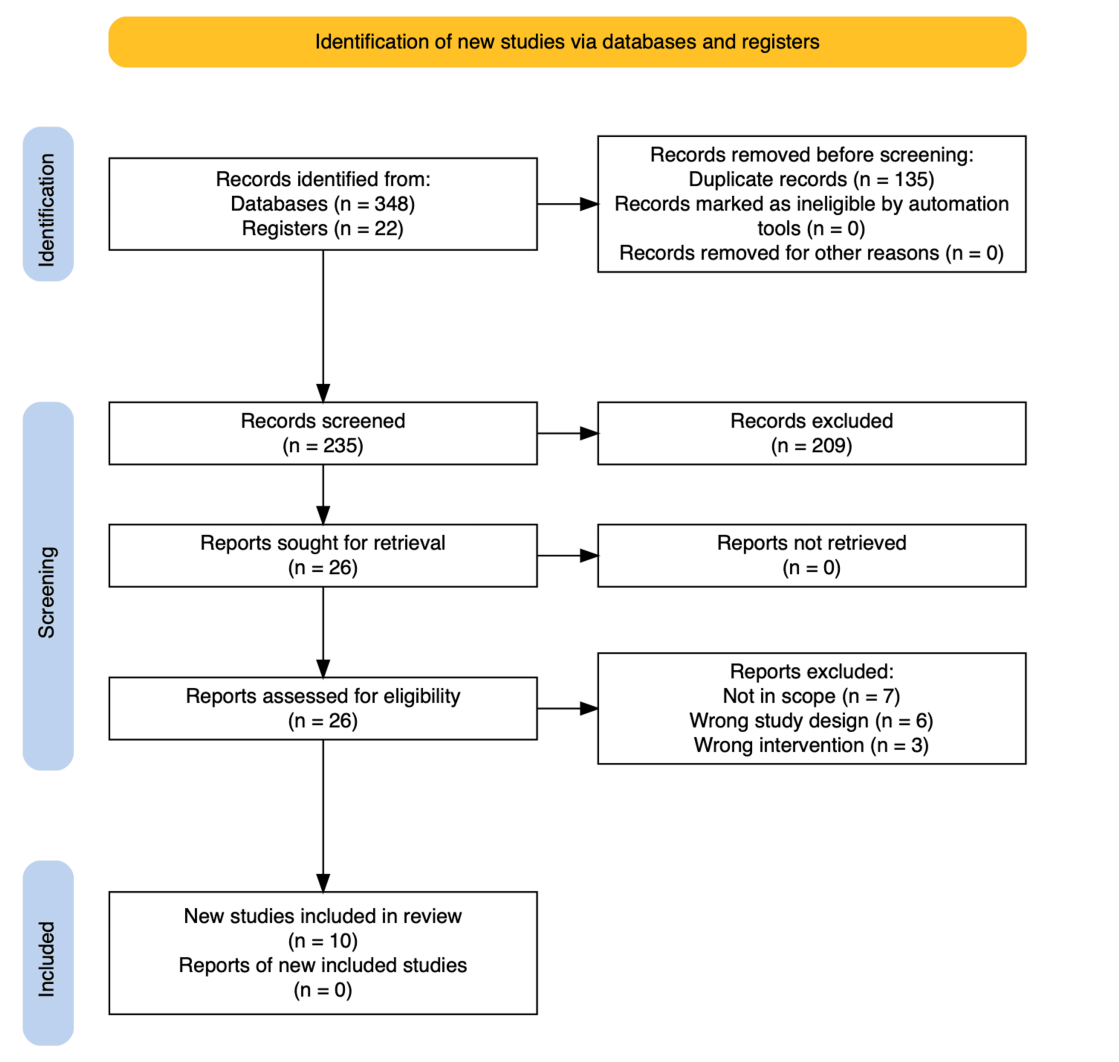
PRISMA flowchart for literature search and study selection n: number of studies; PRISMA: Preferred Reporting Items for Systematic Reviews and Meta-Analyses

The articles were selected in two steps: first, the title and abstract were screened, followed by the complete text. The abstract screening was conducted using a blinded online software with two reviewers. Two hundred and thirty-five matched the original search terms. Twenty-six articles were pending full-text evaluation after removing duplicates and applying the inclusion and exclusion criteria. After a full-text review, a final group of 10 papers was selected for the systematic review (Figure 1). Only randomized clinical trials and prospective intervention studies were assessed because the research group wanted to examine interventions.

The inclusion criteria for articles were randomized controlled trials (RCTs) with an intervention and control group, prospective interventional studies, and completed studies.

The exclusion criteria comprised the following: the unavailability of electronically accessible full-text, publication not in the English language, papers published before 9/09/2009, studies that have yet to be completed, meta-analysis, systematic reviews, and retrospective studies.

Two reviewers performed data extraction on each chosen article using a standardized form. The following information was extracted and entered into a Microsoft Excel spreadsheet: the authors' name, year, title, number of subjects, variables, and findings/conclusion.

Ten articles were reviewed to address the benefits and drawbacks of using dupilumab over the standard of care, ESS, in managing patients with CRS. Table 1 provides an overview of this review.

Results

Gold Standard Treatment for CRS: Functional Endoscopic Sinus Surgery

In 2015, Rotenberg and Pang reported significant positive results from a study exploring the impact of FESS on sleep improvement and nasal obstruction. In a clinical trial study, 53 patients who suffered from CRS were operated on with FESS. Sleep outcomes were measured with the Epworth Sleepiness Scale (EpSS). The EpSS score dropped significantly after surgery (probability (p) < 0.01), which indicates daytime somnolence was a problem preoperatively but normalized postoperatively. Patients’ SNOT-22 scores were measured preoperatively and six months postoperatively, showing a statistically significant improvement (p < 0.01) [9].

Similarly, Alt, DeConde, and their colleagues conducted a multi-cohort pilot intervention study with a population-based sample of 405 adults with CRS and measured QoL indicators following ESS, including sleep dysfunction due to obstructive sleep apnea (OSA). In their pilot study, they explored the variables of sleep quality (p < 0.001), as well as multiple QoL measures, among patients diagnosed with OSA. The researchers aimed to support the premise that many patients with CRS also suffer from OSA and report much lower QoL scores than those without comorbidity. ESS significantly improved QoL scores in CRS patients without OSA (p < 0.001), whereas improvements were less pronounced in those with OSA (p = 0.18). Specifically, the study showed that patients without OSA reported significant reductions in poor sleep prevalence (p < 0.001), whereas no such significant changes were observed in the OSA group (p = 0.18). These findings indicate that treating CRS with ESS can improve QoL outcomes across various domains, but the presence of OSA may limit the extent of these improvements. The study aimed to demonstrate increased QoL scores for patients with CRS who were also assessed and diagnosed with OSA and to highlight the potential impact of treating nasal obstruction through ESS in enhancing sleep and QoL outcomes [10].

Adnane et al. investigated QoL outcomes after FESS surgery for nasal polyposis. Their prospective study was performed with 85 individuals affected by CRSwNP who underwent FESS. Their study examined the rhinosinusitis disability index (RSDI) and the Lund-Kennedy scoring system. There was a significant reduction in pre- and post-op RSDI (p < 0.0001). This finding suggests that while the Lund-Kennedy score is useful for anatomical assessment, it may not reliably predict subjective QoL outcomes following surgery [11].

Yousefi et al. conducted a randomized clinical trial on 30 patients with CRS without nasal polyps. They wanted to measure olfactory outcomes with ESS versus nonsurgical treatment. They conducted their study by dividing the patients into two groups: one underwent ESS, whereas the control group followed a regimen of nasal irrigation four times a day with normal saline. The research group measured the olfactory threshold using the nasal threshold test. The group discovered no difference between the two groups. The mean nasal threshold test in the endoscopic group was 2.79 and 2.67 in the control group (p = 0.930). Their study shows that ESS has no olfactory effect. This highlights that ESS has no negative olfactory impact, a possible complication of sinonasal surgery [12].

Hai et al. conducted a prospective study to determine whether ESS alters bacterial biofilm in patients with CRS. Their study included a total of 28 patients. The study had patients complete an SNOT-20 questionnaire and a visual analog score (VAS) before the surgery and 12 weeks after ESS. The SNOT-20 is similar to SNOT-22 with the exception of two questions. During the procedure, nasal swabs were taken from the middle meatus and 12 weeks post-surgery. The group found a significant difference in pre- and post-op SNOT-20 scores: pre-ESS 2.34 and post-ESS 1.26 (p < 0.01). They found a significant improvement in VAS and nasal endoscopy scores, both reported with p-values (p < 0.01). Regarding biofilm prevalence, patients with biofilm-positive bacteria decreased from 75% pre-surgery to 50% post-surgery, which was a significant reduction (p < 0.05). However, the reduction in biofilm did not significantly correlate with improvements in symptoms or QoL scores (p > 0.05). The results highlight the significant improvement in QoL measures in patients with CRS. ESS reduced the prevalence and strength of bacterial biofilms; however, this reduction was not directly correlated with subjective symptom improvement [13].

DeConde et al. conducted a multi-institutional, comparative effectiveness cohort study to evaluate the effectiveness of ESS versus medical management in improving the cardinal symptoms of CRS. The researchers enrolled 342 subjects into two groups: one (n = 273) underwent ESS, and the other continued their previous medical management of CRS (n = 69). Patients were asked to complete items on the SNOT-22 scoring system. The researchers stated that the four cardinal symptoms of the SNOT-22 were thick nasal discharge, facial pain/pressure, loss of smell/taste, and nasal obstruction. Participants were asked to complete baseline surveys and at least six months post-intervention. The researchers found that the ESS group had significantly higher baseline SNOT-22 scores (53.6 ± 18.8 versus (vs) 44.3 ± 18.9; p < 0.001). Participants in the ESS group were substantially more likely to have improvement in thick nasal discharge, facial pain/pressure, loss of smell/taste, and nasal obstruction, with all p-values reported as <0.001. Participants in the ESS group were also significantly more likely to report complete resolution of thick nasal discharge (p < 0.001), facial pain/pressure (p = 0.002), and nasal obstruction (p = 0.009), with no significant difference in loss of smell/taste (p = 0.270). This study highlights the effectiveness of ESS for the treatment of CRS. However, it is worth noting that the medical management reported in this study does not include dupilumab [14].

Use of Dupilumab in the Management of CRS

In 2019, Bachert et al. conducted a multinational, multicenter, randomized, double-blind, placebo-controlled, parallel-group study of 276 patients and measured the efficacy and safety of dupilumab in the management of CRSwNP. The drug was assessed using measures of nasal polyp score (NPS), polyp size, sinus opacification, and severity of symptoms. At 24 weeks, dupilumab significantly improved nasal congestion/obstruction scores (-0.89, p < 0.0001) and reduced nasal polyp size (-2.06, p < 0.0001) compared to placebo. The Lund-Mackay CT score was -7.44 (p < 0.0001) with dupilumab versus placebo -5.13 (p < 0.0001). Nasopharyngitis, worsening of nasal polyps and asthma, headache, epistaxis, and injection site erythema were more familiar with placebo [15].

Similarly, in 2022, Mullol et al. built on these findings by focusing on olfactory outcomes with dupilumab in CRSwNP patients. Their research group conducted a randomized longitudinal intervention study of 724 patients and measured olfactory outcomes with dupilumab in managing CRSwNP. Loss of smell (LoS) score improved with dupilumab compared to placebo, with a difference of -0.07 (p < 0.0001) at week 24. Dupilumab improved the mean UPSIT (University of Pennsylvania Smell Identification Test ) by 10.54 at week 12 from a baseline of 13.90 (p < 0.0001). The improvements were not affected by CRSwNP duration, prior sinonasal surgery, or comorbid asthma [16].

Expanding on these studies, Haxel et al. conducted a real-world observational study in 2022, exploring the effectiveness of dupilumab compared to omalizumab in managing CRS. Their study of 70 patients examined the effectiveness of dupilumab versus omalizumab. The efficacy was measured through outcomes of polyp score, QoL measures, and olfaction. With both treatments, the polyp score decreased significantly after three months (p ≤ 0.001). QoL parameters, olfaction increased (p ≤ 0.001) and the SNOT-22 score was significantly decreased (p ≤ 0.001). The two treatments had no difference in overall response [17].

In 2019, Jonstam et al. took a different approach by conducting a randomized, placebo-controlled, phase 2 clinical trial on dupilumab, focusing on its effects on type 2 inflammatory biomarkers in nasal secretions and polyp tissue. The drug was assessed by measuring cytokines, chemokines, and IgE levels in nasal secretions and polyp tissue for 16 weeks. With dupilumab, type 2 biomarker concentrations in nasal secretions decreased versus placebo in eotaxin-3 (-30.06 vs. -0.86 pg/mL, p = 0.0008) and total IgE (-7.90 vs. -1.86 IU/mL, p = 0.022). There was also a decrease in type 2 biomarkers in nasal polyp tissues with dupilumab versus placebo for eosinophilic cationic protein (p = 0.008), eotaxin-2 (p = 0.008), eotaxin-3 (p = 0.031), pulmonary and activation-regulated chemokine (p = 0.016), IgE (p = 0.023), and IL-13 (p = 0.031). The study showed that dupilumab statistically significantly reduced inflammatory markers compared to placebo [18].

In 2020, Bachert et al. extended the scope of these studies by examining the broader impact of dupilumab on health-related QoL in patients with CRSwNP. The research group designed a prospective study to investigate the impact of dupilumab on improving health-related QoL (HRQoL) in patients with CRSwNP. The study explored outcomes using the CRS disease VAS, SNOT-22, the 5-dimension EuroQoL (EQ-5D) general health status VAS, and the 36-item Short-Form Health Survey (SF-36) for HRQoL, as well as nasal polyp-related health care resource questionnaires. After 16 weeks, among patients treated with dupilumab, the percentage of those with moderate to severe CRSwNP (VAS >3-10) decreased from 86.2% to 21.4%, while placebo improved from 88.0% to 84.2%. Dupilumab significantly improved HRQoL based on SNOT-22 (p <.001), SF-36, and EQ-5D VAS. Dupilumab also resulted in a statistically significant lower adjusted annualized mean number of sick leave days (0.09 vs. 4.18 with placebo, p = 0.15) [19].

**Table 1:** All reviewed articles list FESS: function endoscopic sinus surgery; p: probability; QoL: quality of life; OSA: obstructive sleep apnea; NPS: nasal polyp score; CRS: chronic rhinosinusitis; ESS: endoscopic sinus surgery; vs: versus; UPSIT: University of Pennsylvania Smell Identification Test; VAS: visual analog score; SNOT-22: Sinonasal Outcome Test

Study Authors	Title	Study Design	Sample Size	Treatment	Key Outcome(s)
Adnane et al. (2015) [11]	Quality of life outcomes after functional endoscopic sinus surgery for nasal polyposis	Prospective study	85	FESS	Significant improvement in QoL post-FESS (p < 0.0001)
Alt et al. (2015) [10]	Quality of life in patients with chronic rhinosinusitis and sleep dysfunction undergoing endoscopic sinus surgery: a pilot investigation of comorbid obstructive sleep apnea	Multi-cohort pilot study	405	ESS	Improved QoL for patients without OSA (p < 0.001)
Bachert et al. (2019) [15]	Efficacy and safety of dupilumab in patients with severe chronic rhinosinusitis with nasal polyps (CRSwNP)	Randomized, double-blind, placebo-controlled study	276	Dupilumab	Significant reduction in NPS and sinus opacification (p < 0.0001)
DeConde et al. (2015) [14]	Investigation of change in cardinal symptoms of CRS after surgical or ongoing medical management	Comparative cohort study	342	ESS vs. medical management (not including Dupilumab)	ESS significantly improved all CRS symptoms except smell (p < 0.001)
Hai et al. (2010) [13]	The effect of endoscopic sinus surgery on bacterial biofilms in chronic rhinosinusitis.	Prospective study	28	ESS	Significant improvement in SNOT-20, VAS and nasal endoscopy score (p < 0.01)
Haxel et al. (2022) [17]	Real-world-effectiveness of biological treatment for severe chronic rhinosinusitis with nasal polyps	Real-world observational study	70	Dupilumab vs. omalizumab	olfaction increased (p ≤ 0.001) and SNOT-22 score was significantly decreased (p ≤ 0.001)
Jonstam et al. (2019) [18]	Dupilumab reduces local type 2 pro-inflammatory biomarkers in chronic rhinosinusitis with nasal polyposis.	Randomized placebo-controlled trial	60	Dupilumab	Significant reduction in type 2 biomarkers (p < 0.05)
Mullol et al. (2022) [16]	Olfactory outcomes with dupilumab in chronic rhinosinusitis with nasal polyps	Randomized longitudinal study	724	Dupilumab	Improved olfactory outcomes and UPSIT scores (p < 0.0001)
Rotenberg and Pang (2015) [9]	The impact of sinus surgery on sleep outcomes	Clinical trial	53	FESS	Improved sleep outcomes (p < 0.01)
Yousefi et al. (2018) [12]	Effect of ESS on the olfactory threshold of patients with CRS without nasal polyps	Randomized clinical trial	30	ESS vs. nasal irrigation	No significant difference in olfactory outcomes (p = 0.930)

Discussion 

CRS significantly impacts patients’ QoL, the condition is multifaceted and requires treatment to be tailored to each patient This systematic review compared the efficacy and limitations of dupilumab and ESS in CRSwNP, focusing on symptom reduction, QoL improvements, and treatment feasibility. The findings of this review suggest that both interventions have distinct advantages and limitations, however, neither emerged as a definitive replacement for the other. Instead, their applications might be complementary and guided by patient-specific factors, disease responsiveness, and treatment goals.

This review highlights Dupixent’s efficacy as a targeted therapy for CRSwNP, this is due to its ability to inhibit interleukin 4 and interleukin 13 signaling, which are pivotal mediators of type 2 inflammation. The studies reviewed in this paper consistently demonstrated improvement in patient-reported outcomes, which include reduction in nasal obstruction, Snot-22, and nasal polyp size. Dupixent improved olfactory outcomes even in patients with long-standing CRSwNP or prior sinonasal surgery, demonstrating its utility across diverse CRS patient populations. Dupixent inhibits the underlying pathophysiology of CRSwNP, directly leading to QoL improvement. However, the high cost of Dupixent and the lack of long-term data are key limitations. Future studies are needed to determine long-term efficacy, cost-effectiveness, and long-term complications.

ESS remains the cornerstone of CRSwNP treatment, especially in patients who fail first-line medical management. This review highlighted its effectiveness in improving key CRS symptoms, such as nasal obstruction, facial pain, and thick nasal discharge, with significant reductions in SNOT-22 scores and other QoL metrics. ESS also facilitates better sinonasal aeration and medication delivery, addressing anatomical and functional contributors to CRS. While ESS demonstrated significant improvements in most QoL domains, its impact on olfaction was less pronounced, particularly in patients without nasal polyps. This shows the need for adjunctive therapies in addressing olfactory dysfunction. Furthermore, the reduction in biofilm prevalence post-ESS, although not directly correlated with symptom improvement, suggests that the procedure exerts additional microbiological benefits that warrant further exploration. ESS also impacted sleep outcomes, although this was not reported/studied in the Dupixent review.

When comparing the two, one crucial factor is the associated cost; according to the Dupixent website, the cost is $3,993.36 United States Dollars (USD) per carton, which contains two 300 mg pens; the suggested dosing for CRSwNP is 300 mg biweekly, which gives a cost of 3,993.36 per month list price [20]. The mean cost of endoscopic surgery for nasal polyps was reported as 14,697 USD [21]. The cost-effectiveness of each depends on the patient's clinical presentation, response to treatment, and long-term needs. For patients with CRSwNP who respond well to Dupixent, the ongoing costs may be justified to avoid surgery. Conversely, ESS provides a definitive intervention, potentially eliminating or reducing the need for long-term pharmacologic management and long-term costs.

Along with this, Dupixent targets inflammation and type 2-driven CRSwNP and offers a non-invasive option for patients who do not want surgery or are not candidates for surgical intervention. However, there is a lack of studies for long-term tolerance, and Dupixent only works as long as the patient is taking the drug. ESS addresses anatomical abnormalities and can provide immediate relief of symptoms; however, it poses a risk for surgical complications.

Head-to-head studies directly comparing these two treatments are lacking, and while duping has shown great promise, more homogeneous studies are needed. The heterogeneity in study designs, patient populations, and outcome measures seen in this review further complicates comparisons. The choice between Dupixent and ESS should be based on each patient, considering disease phenotype, patient preference, comorbidities, and cost. For CRSwNP patients with significant type 2 inflammation, Dupixent may offer a compelling alternative or adjunct to surgery. Conversely, ESS may remain the gold standard for CRSwNP patients with significant anatomical abnormalities or without nasal polyps.

Limitations and future directions

Despite the promising findings from the studies reviewed, notable limitations require consideration. First, many included trials had relatively small sample sizes or were single-center in design, which may limit the generalizability of results. Larger, multicenter trials with standardized methodologies are needed to substantiate the benefits of Dupixent and ESS in broader CRS populations.

Second, the majority of the research centered on CRSwNP, leaving questions about how these interventions perform in patients without nasal polyps, those with diverse comorbid conditions (e.g., OSA, asthma), or individuals from varied demographic backgrounds. Future studies should extend enrollment criteria to capture a broader spectrum of CRS phenotypes and incorporate direct head-to-head comparisons of Dupixent and FESS.

Additionally, the long-term effects of both treatments on QoL, symptom recurrence, and healthcare resource utilization remain underexplored. While biologic therapy with Dupixent has shown encouraging short-term results, there is limited data on whether these outcomes persist once discontinued and potential long-term adverse effects or economic implications. Similarly, although ESS can provide definitive anatomical relief, the durability of its benefits and the possible need for revision surgery warrant further longitudinal evaluation. Although ESS is time-tested, finding new literature focusing on procedural outcomes was difficult.

## Conclusions

In treating CRS, one new asset in managing patients with nasal polyps is Dupixent, which has proven results in addressing type 2 inflammation head-on in a nonsurgical approach. At the same time, ESS remains a key intervention in managing these patients. Nevertheless, ongoing research will be crucial to optimize the sequencing and combination of these therapies. Ultimately, these therapies should be integrated with a personalized approach for each patient who fails first-line treatment. As more studies are conducted, they will refine treatment paradigms and improve the lives of patients affected by this condition.
